# Supplementary material for: Levels and determinants of health literacy and patient activation among multi-morbid COPD people in rural Nepal: Findings from a cross-sectional study
Source: PLoS One. 2020 May 29;15(5):e0233488. doi: 10.1371/journal.pone.0233488 (PMC7259703; doi:10.1371/journal.pone.0233488)
Supplement: Attachment — Submitted filename: Plos One-Editor.docx [file pone.0233488.s002.docx]

Dear Editor, Date: 29/11/2019

I would take this opportunity to thank you and the reviewers for your valuable remarks. We authors have addressed all the remarks and have improved the quality of paper. In particular, we revised our analysis using logistic regression method.

Once again, many thanks for your valuable remarks.

Looking forward to hearing from you.

Warm Regards,

Uday

| Editor remarks |  |
| --- | --- |
| Please clarify scoring and categorization of HQL and PAM in more detail. We used the PAM questionnaire as an instrument to measure Customer Quality in our work as a third dimension to quality. You can find more detail in "Gholipour K, Tabrizi JS, Jafarabadi MA, Iezadi S, Mardi A. Effects of customer self-audit on the quality of maternity care in Tabriz: A cluster-randomized controlled trial. PloS one. 2018 Oct 11;13(10):e0203255." How you categorized PAM result in four level? | Thanks much for sharing the wonderful piece and critical look. We have added the cut-off in the updated document.  **Health Literacy**  The cut-offs to define “high health literacy level” was determined considering upper quartile and the lower two quartiles as “low health literacy level”. This applied to categorize all the five HLQ domains as there was no any standard cut-off.[Line 186-188]  **PAM**  The raw score was calculated using password protected scoresheet provided by Insignia Health and then excel data was imported in SPSS.(21). The raw scores of PAM were converted into four activation levels: (i) Level 1(0.0-47.0): disengaged and overwhelmed, (ii) Level 2(47.1-55.1): becoming aware, but still struggling, (iii) Level 3(55.2-72.4): taking action and, (iv) Level 4(72.5-100.0): maintaining behaviors and pushing further.(21, 22)  [Line 196-200] |
| Provide detailed reference for SPSS as " IBM SPSS Statistics for Windows, version 23 (IBM Corp., Armonk, N.Y., USA)". | We have included in the revised version. |
| Please report regression prerequisite in method section and its handling methods. | Based on reviewer and editor remarks, we checked the assumptions of linear regression and we revised our analysis and have used logistic regression.  The statistical analyses were performed using the Statistical Package for Social Sciences (SPSS 23.00)(25).Participant characteristics were summarised as frequency and percentage. Normality of the data was assessed using Shapiro-Wilk test. We used Chi-Square test for univariate analysis and all the independent variables that had p<0.2 were considered in multivariable regression analysis. Step-wise multivariable logistic regression analysis was used to identify the determinants of poor HL and PA levels and the p-value ≤ 0.05 were considered as significant. |
| How you test the normality of data to use T-test and ANOVA? What test did you used in case the data were not normal? | The Shapiro-Wilks test was used to investigate the normality of the data and we found no violations of regression assumptions. A significance level of p<0.05 for all the independent variables with dependent variables (HLQ domains and PAM) in univariate analysis were considered in multivariable regression analysis. Multivariable linear regression analysis was used to assess associations of the independent variable with scores of HLQ scales and PAM separately. [Page 7] |
| A P-value was duplicated in table 2.  Provide B: Unstandardized coefficients, S.E.: Standard error and Beta: Standardized regression coefficients for variable and model Goodness of Fit in table 3. | Analysis is changed in the revised version. |
| **Reviewer 1** |  |
| Multivariate logistic regression is better statistical analyses in this research than multiple linear regression. It's suggest to analysis with multivariate logistic regression and compare the results. | Dear Reviewer,  Thanks for your suggestion and we have changed our analysis. Much appreciated for this. |
| We know that the conditions used by the Independent t-test and One-way Analysis of Variance (ANOVA) are that the data should conform to the normal distribution and the homogeneity of the variance. Has the author conducted the test? If not, use the rank sum test please.- | Analysis is changed in the revised version.  We used Chi-Square test for univariate analysis and all the independent variables that had p<0.2 were considered in multivariable regression analysis. Step-wise multivariable logistic regression analysis was used to identify the determinants of poor HL and PA levels |
| The Illness Perception scale indicator is not statistically analysed in Tables 1 and 2. Please explain why it included in Table 3 is. 4. Page 13, the authors describe the majority of the participants had a high of illness burden (BIPQ mean 49.83, SD= 8.86), but it is unclear as to the purpose of including this number. | We have included in results section as: The majority of the participants had a high of illness burden (BIPQ mean 49.83, SD= 8.86).  Thank you for very much for your sharp look.  In this revised version we have included it Table1,2 and Table 3. |
| Page 14, the authors do not provide information on whether the overall model was significant or accompanying descriptions of effect size, For example: Square value of R. | Analysis is changed in the revised version.  Models are adjusted for all the included variables in logistic regression. |
| Reviewer 2 |  |
| The authors present a paper addressing the levels and determinants of health literacy and patient activation in multimorbid COPD patients in rural Nepal. Health literacy is a very important subject to reduce the impact of chronic diseases in health systems. The results are relevant to the community chosen for this study. Although the subject is interesting the paper does not read well. There are misspellings throughout the text that should be corrected. In measurement section the text is not clear. What do the authors mean by items? | Thanks much for noting our misspellings. We have worked on the misspellings throughout the paper. Items mean the questions in each domain. |
